# Effectiveness of germinated brown rice on metabolic syndrome: A randomized control trial in Vietnam

**DOI:** 10.3934/publichealth.2020005

**Published:** 2020-01-17

**Authors:** Truong Tuyet Mai, Tran Thu Trang, Tran Thi Hai

**Affiliations:** 1 National Institute of Nutrition, 48B Tang Bat Ho street, Hanoi, Vietnam; 2 Nutrition Department, Faculty of Environmental and Occupational Health, Hanoi University of Public Health, 1A Duc Thang Road, North Tu Liem district, Hanoi, Vietnam

**Keywords:** pre-germinated brown rice, metabolic syndrome, GABA, germinated brown rice, white rice

## Abstract

To treating Metabolic Syndrome (MetS) in the human body by using cooked pre-germinated brown rice (PGBR), a randomized control trial was done in Vietnam. 80 subjects (65.1 ± 3.81 years old) separated into two groups in pairs were assigned a daily intake of 200 g PGBR, and the subjects in the placebo group were kept in normal living ways (consuming white rice). Before baseline and after 3 months of the intervention, anthropometric measurements, blood chemical examinations, a nutrition survey, and physical activity measurements were conducted. The main finding showed that the percentages of patients with MetS in the PGBR group were reduced significantly compared to the placebo group (p < 0.05). Serum HDL cholesterol concentrations were significantly increased from 1.11 (mmol/l) to 1.44 (mmol/l) compared to the placebo group (p < 0.05). The findings suggest that PGBR may affect HDL cholesterol, PGBR might be considered in reducing the risk of MetS in Vietnam.

## 1. Introduction

Metabolic syndrome (MetS) refers to a cluster of metabolic disorders such as hyperglycemia, insuline resistance, type II diabetes mellitus, and hyperlipidemia which cause obesity and cardiovascular diseases [1]. Diet quality is a strong predictor of this syndrome [1],[2]. A diet, which is high-carbohydrate and low fibre, increases directly postprandial levels of blood glucose and insuline, which leads to insuline resistance [3]. Insuline resistance is the cause of type II diabetes mellitus and cardiovascular diseases [4]. Therefore, eating well-quality diets would be well-advised for controlling metabolic syndrome [5]. White rice is the most commonly starchy food staple, especially in developing countries of Asia and Africa [6]. However, its high glycemic index leads to white rice could not be frequent consumption food for diabetes patients [7]. Pre-germinated brown rice (PGBR) may be more benefits than white rice. PGBR, a newly developed type of rice, was made by soaking brown rice kernels in water to slightly germinated them [8]. There are few studies proved the effects of PGBR on the prevention and treatment of metabolic syndrome [9]–[11]. The aim of this study was to investigate the effects of PGBR used to treat metabolic syndrome patients.

## 2. Materials and methods

### 2.1. Settings and subjects study

The protocol of this study was approved by the ethics committee of the National Institute of Nutrition (NIN), Hanoi, Vietnam (number 1656/QĐ-VDD) and also approval from all participants, before recruiting subjects. 80 subjects with the metabolic syndrome (MetS) were recruited in the intervention. All of them live in Bac Ninh City. Bac Ninh City is in the Red River Delta of northern Vietnam, approximately 40 km from the capital, Hanoi. All subjects were fully informed concerning the process of the study, and the study was designed in with the Helsinki Declaration on Human Studies. Eligibility criteria were: age from 55 to 70 years old, live in Bac Ninh City, and were diagnosed having MetS. A subject was defined as having MetS if he/she was any three of the following six factors: waist circumference > 90 cm for men and > 80 cm for women; blood triglyceride level ≥ 1.69 mmol/L; serum HDL-cholesterol concentration < 1.04 mmol/L for men and < 1.3 mmol/L for women; fasting plasma glucose > 6.1 mmol/L; systolic blood pressure ≥ 130 mmHg and/or diastolic blood pressure ≥ 85 mmHg. Exclusion criteria included the following: subjects who were taking medicines that controlled blood glucose or lipid metabolic; subjects who were suffering from serious heart diseases, brain diseases, kidney diseases, liver diseases, or gastrointestinal diseases; subjects who were consuming PGBR daily; subjects who had body mass index (BMI) under 18.5; subjects who were not feeling well after starting the intervention. After screening, 86 subjects met the requirements. During 3 months of the intervention, 3 subjects in each group were excluded because of the individual health status.

The subjects were divided randomly into two groups: a PGBR group (n = 43) and a placebo group (n = 43). The subjects in PGBR group received 1 PGBR capsule (2.0 kg) containing 200 g PGBR/day for 10 days/time, and subjects in the placebo group took 1 capsule (2.0 kg) containing 200 g white rice/day for 10 days/time. The amount of energy, lipid, and protein is almost equivalent to the placebo and PGBR capsules. All subjects were advised to avoid change in their lifestyle, and no subjects were taking any medicines that affected cholesterol levels or body fat reduction during the time of the study.

We measured blood samples at the laboratory of NIN, Hanoi. Blood samples were taken an over-night fast. The PGBR and placebo groups were measured on the same day.

### 2.2. Anthropometric measurements

Weight, height, waist and hip circumferences were measured two times. Bodyweight and height were measured in light clothing and without shoes. BMI was calculated as body weight per height squared (kg/m^2^). Waist circumference was measured mid-way between the lower rib margin and the iliac crest, while hip circumference was measured at the broadest circumference around the buttocks.

### 2.3. Nutrition survey

We used the Semi-Quantitative Food Frequency Questionnaire for a nutrition survey at baseline and completion. Energy and nutrient intake were measured based on the Vietnamese Food Composition Table 2007 [12].

### 2.4. Physical activity survey

We used the structured questionnaire for a physical activity survey at baseline and completion. The levels of physical activity were built based on the Global Recommendations on Physical Activity for Health [13].

### 2.5. Blood sample collection

Blood samples were taken in the morning and after an overnight fast and were measured two times. Fasting glucose was measured by Accu-Chek glucometer. Venous blood samples were kept frozen at 8 °C for analysis: serum total cholesterol, HDL cholesterol, LDL cholesterol, concentrations of insuline, fasting triglyceride.

### 2.6. Blood pressured

Blood pressure was measured by using the mercury sphygmomanometer. All subjects were measured in a quiet, air-conditioned room (the room temperature at 25 °C). The participants had to rest before and during measurements for about 20 min.

### 2.7. Homeostatic Model Assessment for Insuline Resistance (HOMA-IR)

HOMA-IR was calculated using the following formula: fasting insuline (mU/l) × fasting glucose (mmol/l)/22.5. Fasting glucose and insuline were measured using an enzymatic method and chemiluminescence immunoassay, respectively.

### 2.8. Statistical analysis

Data were analyzed by using SPSS for windows 16.0. We calculated the change in percentage for each variable at completion and baseline. All variables of the PGBR and placebo groups were compared using an unpaired *t*-test, and data at baseline and completion were compared using a paired *t*-test. Varibales with confirmed homoscedasticity were compared by Student's *t*-test. *p* values of less than 0.05 were considered statistically significant for all analyses. Mann-Whitney test and Wilconxon test were used in non-normal distribution. Mc Nemar test – non parametrict test – was used on paired nominal data.

## 3. Results

### 3.1. Subjects

Eighty subjects completed the intervention; 3 subjects from the placebo group and 3 subjects from the PGBR group withdrew from the study, due to personal reasons. All subjects completed over 90% of the study process. No subjects in either group had any symptoms related to the intake of test substances.

### 3.2. Nutrition facts for PGBR and placebo capsules

Table 1 shows the amount of energy, protein, lipid, carbohydrate, ash, sodium, and γ-aminobutyric acid (GABA) per 100 g GPBR and per 100 g white rice.

**Table 1.** Nutrition facts per 100 g of PGBR and white rice.

Nutrition facts per 100 g	PGBR	White rice
Energy (kcal)	357	345
Protein (g)	≥ 7	≥ 5
Lipid (g)	≥ 2	≥ 0.1
Carbohydrate (g)	≥ 60	≥ 75
Fibre (g)	3–4	0.5–0.9
Inositol (mg)	≥ 10	-
GABA (mg)	12–20	1–3

### 3.3. Baseline characteristic

Table 2 shows the baseline parameters of both groups. There were no significant differences in parameters for age, sex, education levels, height, weight, BMI, abdominal circumference, systolic blood pressure, diastolic blood pressure, fasting glucose, insuline, HOMA-IR, total cholesterol, triglyceride, LDL cholesterol, HDL cholesterol, percentage of constipation, physical activity levels, diet incides between the PGBR and the placebo groups.

**Table 2.** Baseline characteristics of PGBR and placebo groups.

		Total sample	PGBR group	Placebo group
N		80	40	40
Age(y)		65.1 ± 3.81	65.2 ± 3.78	65.0 ± 3.85
Sex				
	Male	16 (20%)	8 (20%)	8 (20%)
	Female	72 (80%)	36 (80%)	36 (80%)
Education				
	Primary and secondary	38 (47.5%)	18 (45.0%)	20 (50.0%)
	High school	31 (38.7%)	16 (40.0%)	15 (38.0%)
	Colleges/University/Graduate School	11 (13.8%)	6 (15.0%)	5 (12.0%%)
Occupation				
	Workers/Employees	14 (17.5%)	8 (20%)	6 (15.0%)
	Retirement	46 (57.5%)	20 (50.0%)	26 (65.0%)
	Business	12 (15.0%)	7 (17.5%)	5 (12.5%)
	Housewife	8 (10.0%)	5 (12.5%)	3 (7.5%)
Height (cm)		156.7 ± 5.3	156.5 ± 5.6	156.9 ± 6.3
BMI (kg/m^2^)		25.7 ± 2.2	25.9 ± 2.4	25.5 ± 2.3
Abdominal circumference (cm)				
	Male	92.9 ± 2.4	93.2 ± 3.1	92.6 ± 2.3
	Female	87.6 ± 3.1	88.1 ± 4.6	87.1 ± 3.2
Systolic blood pressure (mmHg)		124.8 ± 15.4	125.0 ± 15.7	124.7 ± 16.5
Diastolic blood pressure (mmHg)		81.9 ± 12.4	82.4 ± 12.2	81.7 ± 13.6
Fasting glucose (mmol/l)		6.4 ± 0.8	6.4 ± 1.2	6.3 ± 0.9
Insuline (mU/l)		9.5 ± 5.4	9.6 ± 6.0	9.4 ± 5.6
HOMA-IR		2.7 ± 1.7	2.7 ± 1.9	2.6 ± 1.8
Total cholesterol (mmol/l)		5.5 ± 0.8	5.5 ± 1.0	5.6 ± 0.9
Triglyceride (mmol/l)		2.4 ± 1.4	2.4 ± 1.6	2.3 ± 1.3
LDL cholesterol (mmol/l)		3.0 ± 0.8	3.0 ± 1.2	3.0 ± 0.6
HDL cholesterol (mmol/l)		1.12 ± 0.2	1.11 ± 0.2	1.12 ± 0.3
Constipation status (%)		22 (27.5%)	12 (30.0%)	10 (25.0%)
Physical activity levels				
	Moderate	56 (70.0%)	28 (70.0%)	27 (67.5%)
	Vigorous	9 (11.3%)	4 (10.0%)	5 (12.5%)
	Intensity	38 (47.5%)	18 (45.0%)	20 (50.0%)

### 3.4. Physical characteristics

In Table 3, we indicate the anthropometrics and blood pressures at all intervention times. No significant difference was observed between the placebo and PGBR groups in weight, BMI, systolic blood pressure or diastolic blood pressure. There were significant reductions in abdominal circumferences of both sexes of the PGBR group between baseline and completion (p < 0.05).

**Table 3.** Physical characteristics throughout the intervention.

		Baseline	End
Weight (kg)			
	PGBR	63.5 ± 8.2	61.8 ± 8.6
	Placebo	62.1 ± 7.9	62.6 ± 8.4
BMI (kg/m^2^)			
	PGBR	25.9 ± 2.4	25.0 ± 2.3
	Placebo	25.5 ± 2.3	25.8 ± 2.6
Abdominal circumference in male (cm)			
	PGBR	93.2 ± 3.1	92.1 ± 3.7 *^a^*
	Placebo	92.6 ± 2.3	92.7 ± 2.6
Abdominal circumference in female (cm)			
	PGBR	88.1 ± 4.6	86.2 ± 5.8 *^a^*
	Placebo	87.1 ± 3.2	86.2 ± 4.0 *^a^*
Systolic blood pressure (mmHg)			
	PGBR	125.0 ± 15.7	120.1 ± 16.4
	Placebo	124.7 ± 16.5	121.9 ± 13.2
Diastolic blood pressure (mmHg)			
	PGBR	82.4 ± 12.2	74.6 ± 11.1
	Placebo	81.7 ± 13.6	77.3 ± 15.3

^*a*^ *p < 0.05, compare to baseline, paired t-test*.

### 3.5. Blood biochemical parameters

Table 4 shows the results of blood tests at month 3 of the intervention. No significant difference was observed between the placebo and PGBR groups in fasting glucose, insuline, HOMA-IR, total cholesterol, LDL cholesterol, triglyceride. HDL cholesterol, and LDL-C/HDL-C ratio were significantly different between the placebo and PGBR groups. Both groups were significantly different in insuline when comparing baseline and completion. Fasting glucose, HOMA-IR, of the PGBR group showed a significant reduction from baseline to completion.

### 3.6. Energy and nutrient intakes and effects on patients with MetS

Table 5 shows the amount of energy and nutrient intake of both groups at baseline and completion; there were no significant differences in energy, protein, carbohydrate, lipid, and fiber between the PGBR and placebo groups at baseline or completion. Fibre was significantly different between the placebo and PGBR groups. Fibre of the PGBR group also showed a significant increase from baseline to completion. No significant difference was observed between placebo and PGBR groups in the physical activity level. At the end of the intervention, there were significant differences in the percentage of patients with MetS between placebo and PGBR group.

**Table 4.** Blood test results throughout the intervention.

		Baseline	End
Fasting glucose (mmol/l)			
	PGBR	6.4 ± 1.2	5.7 ± 0.6 *^a^*
	Placebo	6.3 ± 0.9	6.2 ± 0.9
Insulin (mU/l)			
	PGBR	9.6 ± 6.0	8.3 ± 4.6 *^a^*
	Placebo	9.4 ± 5.6	8.9 ± 4.1 *^a^*
HOMA-IR			
	PGBR	2.7 ± 1.9	1.5 ± 0.9 *^a^*
	Placebo	2.6 ± 1.8	2.4 ± 1.5
Total cholesterol (mmol/l)			
	PGBR	5.5 ± 1.0	5.2 ± 0.9
	Placebo	5.6 ± 0.9	5.5 ± 0.9
HDL cholesterol (mmol/l)			
	PGBR	1.11 ± 0.2	1.44 ± 0.4^¥, *t*^
	Placebo	1.12 ± 0.3	1.11 ± 0.3
LDL cholesterol (mmol/l)			
	PGBR	3.0 ± 1.2	2.9 ± 0.8
	Placebo	3.0 ± 0.6	3.0 ± 0.9
LDL-C/HDL-C ratio			
	PGBR	2.9 ± 1.1	2.3 ± 0.6^¥, *t*^
	Placebo	2.8 ± 0.9	2.9 ± 0.9
Triglyceride (mmol/l)			
	PGBR	2.4 ± 1.6	2.0 ± 1.4
	Placebo	2.3 ± 1.3	2.2 ± 0.9

^*a*^ *p < 0.05, compare to baseline, paired t-test;* *^¥^ p < 0.05, compared with the control group, Mann-Whitney test; ^t^ p < 0.05, compare to baseline, Wilconxon test*.

**Table 5.** Energy and nutrient intakes.

		Baseline	End
Energy (kcal)			
	PGBR	1260.4 ± 387.7	1263.1 ± 401.0
	Placebo	1264.0 ± 337.9	1271.8 ± 331.0
Carbohydrate (g)			
	PGBR	220.4 ± 79.9	221.3 ± 72.1
	Placebo	225.6 ± 78.2	227.6 ± 78.2
Protein (g)			
	PGBR	51.0 ± 18.6	51.3 ± 14.9
	Placebo	50.1 ± 12.8	50.0 ± 13.4
Lipid (g)			
	PGBR	25.0 ± 10.7	24.9 ± 9.0
	Placebo	24.5 ± 12.7	25.4 ± 13.8
Dietary Fibre (g)			
	PGBR	5.3 ± 2.0	9.6 ± 1.8*^, *a*^
	Placebo	5.2 ± 1.8	5.4 ± 1.9
Percentages of MetS reduction (%)			
	PGBR	0	30 (12)^Φ, γ^
	Placebo	0	10 (4)

* p < 0.05, compared with the control group, t-test; ^a^ p < 0.05, compare to baseline, paired t-test; ^Φ^ p < 0.05, compared with the control group, χ2 test; ^γ^ p < 0.05, compare to baseline, χ2 Mc Nemar test.

## 4. Discussion

The study examined the effects of PGBR on patients with MetS in 3-month-intervention in Vietnam. The effects of PGBR on MetS patients were shown in the reduction significantly of MetS percentages in the PGBR group compared to the placebo group. Another intervention in Japanese males also showed that the increases significantly in glucose were lower after the ingestion of brown rice than after the ingestion of white rice [14]. In the present study, abdominal circumferences in the PGBR group was significantly reduced after the 3-month intervention, while those in the placebo group were unchanged with no differences in physical activity levels. Similar to our results, waist circumference (90.3 ± 10.3 cm) and systolic blood pressure (134 ± 13 mmHg) of Japanese males were also lower at the end of the 8-week brown rice diet period [14]. It might prove to be the case that PGBR reduces waist circumference over longer periods of time. Improvement in lipid metabolism and reduction of the abdominal circumference were observed in some studies that have been conducted with Taiwanese [8] and Vietnamese [9] subjects by replacing white rice with PGBR in two of the subjects' meals per day. However, the effect of PGBR on body weight and BMI are also not clear in present intervention.

In the present intervention, MetS definition of the National Cholesterol Education Program Treatment Panel III – Adults: 3^rd^ Report (NECP ATP III) was used to define patients. MetS was diagnosed when patients have ≥ 3 of 5 following criteria which are about waist-line, triglycerides, HDL-cholesterol, fasting blood glucose, and blood pressure. Thus, although only HDL-cholesterol concentrations were significantly increased from 1.11 (mmol/l) to 1.44 (mmol/l) compared to the placebo group, the number of patient with MetS was reduced from 40 patients to 28 patients. Then the percentages of this reduction were higher significantly compared to the reduction of the placebo group (from 40 patients to 36 patients). Moreover, for triglyceride and blood pressure, significant differences between groups were not observed at endpoint. However, in PGBR group, abdominal circumference in both male and female, and fasting glucose decreased significantly compared to its baseline. Future research with longer time intervention is needed.

Our main findings were that PGBR is correlated with HDL cholesterol during comparison between the PGBR and placebo group. Several interventions showed LDL cholesterol in the PGBR group was significantly reduced after the 6-month intervention[11] and after 12-week intervention [15] while non-significant finding was showed in our study. Meanwhile, HDL cholesterol in the PGBR group was significantly increased after the 3-month intervention compared to placebo group and baseline. Fish – Rich diet should be a reason. Omega 3 from fish meat including α-Linolenic acid, eicosapentaenoic acid (EPA), and docosahexaenoic acid (DHA) has been suggested to be similar to that of fibric acids. EPA and DHA, as well as fibric acids, stimulate the β-oxidation of themselves or of other fatty acid substrates, by this mechanism, also elicit peroxisomal proliferation. Thus, polyunsaturated fatty acids may lead to an increase in the HDL cholesterol concentrations. Further researches need to be discovered this different result.

Although the difference of BMI was not significant, reductions significantly of fasting glucose and insuline compared to baseline showed that white rice may be a major cause of diabetes mellitus [16],[17], especially for East Asians, such as Vietnamese who take about 70% of their energy from rice [18]. In previous study, effects of PGBR on fasting glucose was proved by bioactives (γ-aminobutyric acid (GABA), acylated steryl glycoside, oryzanol, and phenolics) involve in PGBR's downregulation gluconeogenic genes (*Fbp1* and *Pck1*) [19].

In this study, the fibre concentration of PGBR group was significantly different from placebo group and baseline while energy, carbohydrate, protein, and lipid were no differences. Fiber slows carbohydrate digestion and glucose absorption. In another intervention (2014), PGBR contains a higher amount of fiber help control blood glucose and lipid concentration in Vietnamese women with Impaired Glucose Tolerance [9]. Effects of fiber to prevent and treatment MetS and diabetes mellitus were also proved in several interventions [20],[21]. However, during the 3-month intervention, percentages of constipation among participants were decreased (the data not shown). This situation may cause a reduction in vegetable consumption's subjects. Thus, the decrease of vegetable intake in PGBR group should be a confounder.

## 5. Conclusion

The results of the present study suggest that PGBR benefits of MetS patients, and may provide long-term benefits of diabetes mellitus. Further studies with longer durations of intervention are warranted to examine the effects of substituting PGBR with white rice on MetS for future cardiovascular and blood glucose diseases.
